# Feasibility of a Multicomponent Frailty Intervention During Post-Acute Rehabilitation in Skilled Nursing Facilities

**DOI:** 10.1111/jgs.70497

**Published:** 2026-05-07

**Authors:** Sandra Shi, Kylee Maclean, Alex Wolfe, Yuchen Liu, Innokentiy Bakaev, Thomas Travison, Dae Hyun Kim

**Affiliations:** 1 Marcus Institute for Aging Research, Hebrew SeniorLife, Boston, Massachusetts, USA; 2 Division of Gerontology, Department of Medicine, Beth Israel Deaconess Medical Center, Boston, Massachusetts, USA

**Keywords:** frailty, post-acute care, skilled nursing facilities

## Abstract

**Background:**

Although over 80% of older adults admitted to skilled nursing facilities (SNFs) for post-acute care are frail, it is unclear whether frailty interventions are feasible in this setting. We piloted a multicomponent frailty intervention in a post-acute SNF population.

**Methods:**

We recruited community-dwelling adults ≥ 65 years old from 2 SNFs (08/2023–10/2024). We excluded those who had a feeding tube, did not have an oral diet, had chronic kidney disease (CKD) stage 4 or worse, could not consent, or were non-English-speaking. We measured demographics, comorbidities, frailty index (FI), and functional status on admission. The intervention consisted of individualized exercises and ≥ 15 g of protein supplementation within 30 min of exercise. Sessions were offered ≥ 5 times a week in addition to regular therapy. Our primary outcome was feasibility of recruitment, defined by the proportion of eligible participants enrolled.

**Results:**

Of 515 admissions screened, 147 were eligible and 50 enrolled (50/147 = 34.0% of eligible; 50/515 = 9.7% of screened). Mean age was 81.2 years (SD: 7.7), 34 (68%) were female, and mean prehospitalization frailty index (FI) was 0.32 (SD: 0.08). Mean SNF length of stay was 16.2 days (SD: 7.7). Overall, 55.3% of sessions offered were completed. Forty participants (80.0%) were discharged to the community, 4 (8.0%) were re-hospitalized, 4 (8.0%) transitioned to long-term care, and 2 (4.0%) had unknown discharge disposition. Eleven participants (22%) withdrew during the SNF stay. No serious adverse events occurred. Among completers (*n* = 34), descriptive improvements from admission to discharge included grip strength +1.4 kg (SD: 3.2), Modified Barthel Index +15.9 (SD: 16.8), AM-PAC Basic Mobility +3.6 (SD: 4.2), and PROMIS Physical Health +3.7 (SD: 7.3).

**Conclusions:**

Delivering a multicomponent frailty intervention in post-acute SNFs is safe, feasible, and acceptable to older adults with frailty. These pilot data support larger trials to test efficacy and scalability.

## 1 | Background

The post-hospitalization period is a critical window of opportunity for older adults to recover from acute medical problems, deconditioning, and functional decline. After acute hospitalization, 1 in 5 older adults receives post-acute rehabilitation at a skilled nursing facility (SNF) [1]. Unfortunately, fewer than half are discharged to the community, with many re-hospitalized, transitioned to long-term nursing home care, or “rehabbed to death.” [2] Most older adults presenting for post-acute SNF care have frailty, a state of increased physiological vulnerability to stressors [3–5], increasing risk of poor health outcomes including disability and institutionalization [6, 7]. Given its prevalence and impact in this setting, there is a critical need for SNF rehabilitation interventions to address frailty.

Rehabilitation structures in the SNF setting have been designed for healthy adults recovering from acute illness or surgical problems, with limited focus on underlying frailty [8, 9]. For example, rehabilitation may be offered as a single daily session lasting up to an hour, 3–5 times each week. However, frail persons have limited endurance and may not be able to tolerate extended therapy sessions, leading to limited rehabilitation therapy time and delayed post-acute recovery [10–12]. However, ‘fragmented dosing’ or additional therapy given in a separate session may be beneficial. Additionally, protein supplementation plays a key role in addressing protein-calorie malnutrition, which affects up to 50% of the SNF population [13, 14]. Although the anabolic effects of protein supplementation are maximized when consumed immediately after exercise [15], supplement intake in SNFs is often low and during meals rather than timed with therapy. Thus, frail patients rarely reap the synergistic benefit of protein timed with exercise. Altogether, the potentially limited rehabilitation and poor timing of nutritional supplementation are missed opportunities to address frailty in older adults.

Community-based multicomponent interventions incorporating exercise and nutritional supplementation have successfully restored function in frail older adults [16–18]. In the SNF setting, an interdisciplinary team consisting of rehabilitation therapists, dieticians, and medical teams provides comprehensive patient care on a day-to-day basis, creating an ideal environment for multicomponent interventions. Tailoring and adapting frailty-focused interventions to the post-acute SNF setting may increase the likelihood of functional recovery and promote independence. However, concerns exist regarding the safety and tolerability of additional rehabilitation in the post-acute setting, particularly among frail and vulnerable populations. Historically, there have also been concerns regarding inappropriate rehabilitation in the setting of little recovery potential [19]. Thus, the question remains whether frail older adults can benefit from multicomponent frailty interventions that include additional exercise in the SNF setting. Here, we aimed to test the feasibility of (1) recruiting frail older adults and (2) implementing a multicomponent frailty intervention in the SNF setting. The frailty intervention was delivered in addition to usual care during a post-acute SNF admission.

## 2 | Methods

### 2.1 | Recruitment

Between August 2023 and October 2024, adults admitted to two SNFs in the Greater Boston Area were screened for eligibility through electronic medical record review. Inclusion criteria were (1) aged ≥ 65 years, (2) community-dwelling prior to hospitalization, and (3) at least mild frailty or worse. The recruitment strategy aimed to identify patients early in the SNF admission without reliance on physician documentation or formal frailty diagnoses. Thus, we defined mild frailty for recruitment by the Clinical Frailty Scale and operationalized it with a validated classification tree [3, 20]. An initial inclusion criterion of inpatient hospitalization for at least three nights was later removed to reflect a growing population of patients admitted under a waiver by their insurance plans. Exclusion criteria included: non-English-speaking, receipt of parenteral nutrition, inability to take an oral diet, Chronic Kidney Disease (CKD) stage 4 or worse, a noncommunity discharge plan at SNF admission (e.g., transition to hospice), inability to consume intervention products (e.g., allergies to supplement ingredients), inability to provide consent to study procedures, or clinician refusal.

In Massachusetts, an “activated” Health Care Proxy indicates that a physician has determined the patient lacks decisional capacity, thereby delegating clinical decision-making authority to a designated Health Care Agent. Our protocol required participants to provide informed consent directly. Consequently, individuals with an activated Health Care Proxy were ineligible for participation and met exclusion criteria by definition. This was determined primarily upon electronic health record review, and included under “clinician refusal” in our protocol to reflect that this was determined by the primary clinical team, and not a research capacity assessment.

A member of the research team approached participants within 72 h of SNF admission. Given the high prevalence of cognitive impairment in the SNF population, a brief assessment was performed to confirm capacity to consent to research (Figure S2). This assessment has previously been used in the SNF setting [21], and is distinct from clinical assessment of capacity or other cognitive assessments which were done as part of study measurements as detailed below. The Advarra Institutional Review Board approved this study. All participants provided written informed consent.

### 2.2 | Intervention

The rehabilitation component of the intervention was based on protocols treating frailty in community settings [16, 17, 22], modified and tailored with input by physical therapy team at the SNFs. The goal was to allow the frailty intervention to complement ongoing treatment provided by the primary treating rehabilitation team, while incorporating key hallmarks of effective frailty interventions, including moderate-intensity resistance, balance, and endurance, with synergistic protein supplementation. Up to three exercises, of seven possible options (Figure S1), were selected by the participant’s primary treating physical therapist upon study enrollment, then updated on a weekly basis. Thus, intervention exercises were in addition to their usual care and standard rehabilitation during their stay. If therapy services were terminated prior to SNF discharge, the research team continued the last set of recommended exercises, throughout the duration of the SNF stay.

A personal care assistant (PCA) or trained research assistant supervised the individualized session daily, up to five times per week, 7 days a week. The weekly intervention exercises specified a target number of reps/sets for each exercise, except for walking which used distance. A total duration/min was not specified. Each session was offered at a time that did not interfere with usual care, typically from 1 to 5 pm. Staff received training through a minimum of two group-based instructional sessions that included exercise demonstrations. In addition, the PI and SNF therapists ensured fidelity to the intervention protocol and safety by observing how research staff administered the intervention to participants on at least one occasion. Their intervention roles were limited to supervision and monitoring for safety, but no physical assistance was provided during exercises. During each session, patient safety was monitored, including patient-reported symptoms and vital signs (blood pressure, heart rate, respiratory rate, and oxygen saturation) before and after the session.

Participants were concurrently offered a commercial nutritional supplement containing at least 15 g of protein with each session [14, 23, 24], timed within 30 min for maximal anabolic effect. Supplements were selected with input from registered dieticians and speech language pathology therapists to ensure alignment with dietary restrictions to accommodate modified diet textures. Options were then individualized according to participant preferences during the study. To allow for flexibility to participant tastes and preferences, we included a range of commercially available supplements, some of which had up to 20 g of protein.

As a feasibility pilot our primary goal was to optimize feasibility and implementation. Rather than being introduced at prespecified endpoints, observations and protocol adaptations were implemented responsively as they arose during the course of the study. For instance, when available protein supplements could not accommodate participant needs (e.g., kosher diet), the study team sought alternative commercially available products that could still meet all protocol-defined requirements. Ultimately supplements varied by flavor, texture (e.g., bars, drinks, gelatin), and source (e.g., plant based, whey protein). A full list of protocol adaptations is presented in Table 1.

The Patient Priorities Care module was introduced in January 2024, at the study midpoint. Many participants had very limited participation in exercise sessions, particularly early in admission, often reporting fatigue. However, they continued to request visits and expressed a desire to remain in the study. Additionally, research staff also reported that participants often asked research staff to communicate concerns to clinical staff. Accordingly, the protocol was modified to include a standardized module to formally collect and share patient care preferences with clinical staff. All participants were offered the opportunity to complete a self-guided and validated Patient Priorities Care module, designed to help patients and caregivers identify health priorities and manage health problems [25, 26]. Completion of the module was optional, and participants could choose whether to share their health priorities summary with their clinical team or others.

### 2.3 | Measurements

At baseline, an in-person assessment was conducted to establish the prehospitalization deficit-accumulation FI (range: 0–1), based on self-reported function and ability to perform physical tasks during the 2 weeks before the preceding acute hospitalization [27]. This was based on prior work, which established the feasibility of measuring prehospitalization frailty in post-acute SNF patients using an FI. The FI included 27 comorbidities, 7 Activities of Daily Living (ADLs), 7 instrumental activities of daily living (IADLs), as well as 8 Nagi and Rosow-Breslau physical tasks [28, 29]. Prehospitalization frailty was thus categorized using standard cutpoints [30, 31]: nonfrail (FI: < 0.25), mild frailty (FI: 0.25 to < 0.35), moderate frailty (FI: 0.35 to < 0.45), and severe frailty (FI: ≥ 0.45). Comorbidities and medical history were determined from the electronic medical record, including age, sex, race/ethnicity, and admission type (medical vs. surgical). Cognition in the SNF was assessed with the Mini-Cog test, but excluded from the prehosptialization FI assessment [32, 33].

Polypharmacy was defined as having five or more scheduled medications, based on the medication list from the SNF EHR. We also recorded whether participants were prescribed high-risk medications, including opioids, benzodiazepines, and antipsychotics. Delirium was assessed using a 3-Minute Diagnostic Assessment for Delirium (3D-CAM) [34]. Pre-SNF hospitalization characteristics (e.g., length of stay, ICU admission), and code status were extracted from medical records via the SNF’s electronic health record (EHR). As there is no integration between the SNF EHR and hospital/regional health records, details of hospitalization were only available from records provided by the hospital upon transfer (i.e., the printed discharge summary, which is scanned into the SNF’s EHR).

At baseline, weekly, and SNF discharge we measured rehabilitation metrics commonly used to predict future functional status, including Activity Measure for Post-Acute Care (AM-PAC) score (basic mobility and daily activities—range: 6–24 with higher scores indicating better function) [35], modified Barthel Index (range: 0–100, higher scores indicate better function) [36], grip strength, and gait speed. Grip strength (kg) was measured with a Jamar dynamometer, reported as the mean of two trials in the dominant hand. Gait speed (m/s) was measured over four meters and reported as the mean of two trials, with assistive devices allowed if needed. Lastly, we measured quality of life with the Patient Reported Outcome Measurement Information System (PROMIS) Global Health short form v1.2 (standardized score with mean 50 and standard deviation [SD] 10, higher is better) [37].

Study participation in sessions was defined as either attempted (participant was offered session and engaged in at least one component), refused (participant declined to participate), or not available (e.g., conflicting medical appointments). If a participant was approached by a trained PCA or research assistant and asked to participate in an intervention session, we counted that as an offered session. These included sessions offered to participants who later withdrew. Thus, it was possible for participants to enroll in the study and never receive a single intervention session. The proportion of the supplement consumed was recorded as a percentage and then categorized (0%—None, 25% or 50%—Partial, 75% or 100%—Most or All). We recorded the total time to complete each session in minutes. If a session was not completed, we recorded the reason (e.g., pain, chest pain, shortness of breath, nausea/vomiting, dizziness, unstable vital signs, participant request, or other).

Adverse events were monitored by trained research staff, with serious adverse events defined as any event resulting in inpatient hospitalization, significant disability, prolongation of rehabilitation stay (i.e., any extension or delay of planned discharge date by the clinical team), or requiring medical or surgical intervention to prevent hospitalization. All adverse events were reviewed by the PI, who reviewed the clinical team’s notes and EHR, to determine relation to the intervention. All events were also reviewed by the study’s safety officer.

### 2.4 | Outcomes

Our primary outcome was feasibility, defined by the proportion of eligible participants who enrolled. Based on prior work recruiting frail older adults in SNF settings, we targeted a recruitment rate of 30% of eligible patients [21, 38]. Secondary outcomes included dropout rates after enrollment and the proportion of sessions that participants attempted, targeting at least 50% of sessions. We also measured acceptability, defined by participant feedback by exit survey, which included satisfaction with overall program, program components, as well as Net Promoter Score [39]. We assessed changes in physical performance measures, including gait speed, grip strength, AMPAC score (basic mobility and daily activities), modified Barthel Index, and PROMIS score. We measured a PT composite score and OT composite score (8 items each, 7 item scale from dependent [1] to independence [7]) from standardized clinical assessments as documented in the EHR, equivalent to functional measures used in the Minimum Data Set [40]. All study data were collected and managed using REDCap electronic data capture tools hosted at Hebrew SeniorLife.

### 2.5 | Statistical Analysis

Participant demographics were summarized using means and standard deviation (SD) or median and interquartile range (IQR), as appropriate. Participation and acceptability were examined by prespecified subgroups of frailty and cognitive impairment. We also calculated the proportion of participants who were satisfied with the intervention from exit surveys done prior to SNF discharge. In exploratory analyses, we examined changes in functional status among participants with discharge physical assessments and converted PROMIS raw scores into physical and mental health-related quality of life *T*-scores. As a pilot feasibility study, we sought to identify objective performance measures that might capture functional change over the course of an SNF stay. Accordingly, we describe both observed changes in these measures and data missingness. No formal statistical testing was done for these measures, as they are intended to be descriptive.

## 3 | Results

**FIGURE 1 |:**
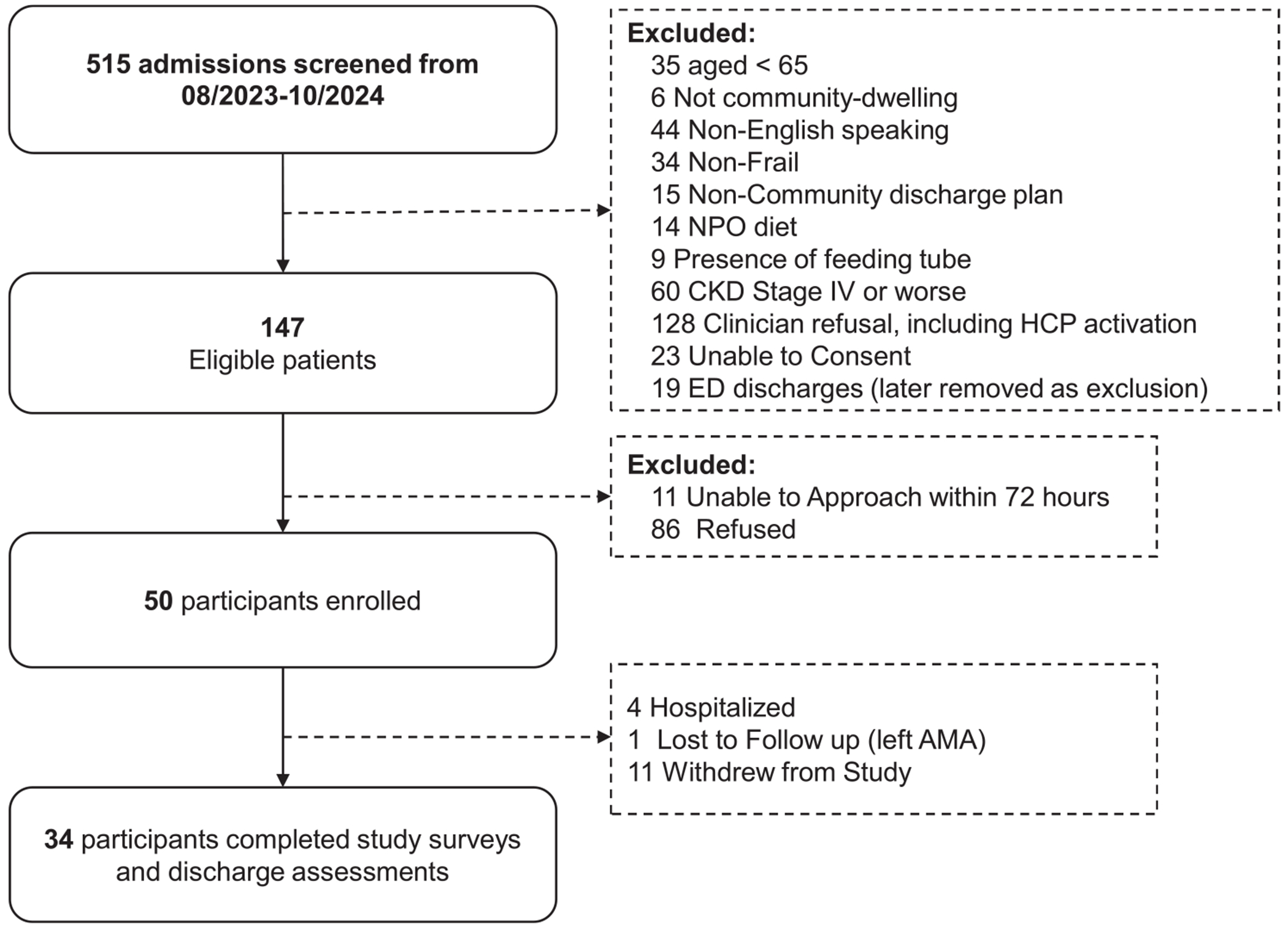
Screening and recruitment for overall study. 515 skilled nursing facility admissions screened between August 2023 and October 2024. Eligible patients were approached within 72 h of admission. Overall, 147 patients were eligible, 50 were enrolled, and 34 completed the study. Individuals with an activated Health Care Proxy, indicating a physician determination of impaired decisional capacity, were therefore excluded and categorized as clinician refusals based on electronic health record review. A brief assessment was used to confirm research consent capacity, which was distinct from clinical capacity determinations. Abbreviations: CKD, Chronic Kidney Disease; ED, Emergency Department; HCP, Health Care Proxy; NPO, Nothing per Oral.

Of 515 screened patients, 147 were eligible, and 50 (34% of eligible, 9.7% of screened) were enrolled (Figure 1). The most common reason for exclusion was clinician refusal, including health care proxy activation (*n* = 128), followed by CKD Stage 4 or worse (*n* = 60). The mean age was 81.2 years (SD: 7.7), and 34 (68.0%) were female (Table 2). Mean length of stay in hospital prior to SNF admission was 7.9 days (SD: 8.1), with 6 participants (12%) having had an ICU admission during hospitalization. The mean baseline FI was 0.32 (SD: 0.08), with 20 (40%) having mild frailty and 19 (36.0%) having moderate frailty or worse. On average, participants reported limitations in 2 ADLs (SD: 1.6) and 3.4 IADLs (SD: 1.5). The most common comorbidities were hypertension 34 (68%), anxiety 16 (33%), anemia 16 (33%), and coronary artery disease 14 (28%). Upon admission, 47 (94%) participants had polypharmacy, 3 (6%) had delirium, and 33 (67%) were full code.

Of the 50 participants enrolled the mean length of stay at SNF was 16.2 days (SD: 7.7). Ultimately 40 (80.0%) were discharged to the community, 4 (8.2%) were re-hospitalized, 4 (8.2%) were discharged to long-term care, and 2 (4.0%) were lost to follow-up. 11 (22%) participants withdrew from the study during their SNF stay, with most of those occurring in the first half of the pilot (36% study withdrawal for the first 25 participants, 8% withdrawal for the latter 25). Two participants reported nausea, which resolved with no intervention. One participant withdrew due to nausea. The participant was subsequently diagnosed with influenza and ultimately re-hospitalized. No severe adverse events related to the study occurred.

Offered intervention sessions were completed 55.3% of the time, lasting an average of 18.1 min (SD: 8.8) per session, with participants refusing 28.3% of sessions and not available for 16.4%. Participants were offered a mean of 6.6 exercise sessions during their SNF stay, of which a mean of 3.6 sessions were received. Among participants who completed the study, the mean numbers of sessions offered and received were 8.4 and 4.9, respectively. The most frequently selected exercises were sitting quad sets (70.5% of all regimens) and reverse fly/shoulder resistance band (45.5% of all regimens). The reverse fly/shoulder resistance band exercise was most frequently completed (29.5% of all sessions). The overall frequency of exercise selection and completion is presented in Table S1. Session completion frequency seemed to vary by overall study status (Did not complete study: 31.1% vs. Completed: 59.2%).

**FIGURE 2 |:**
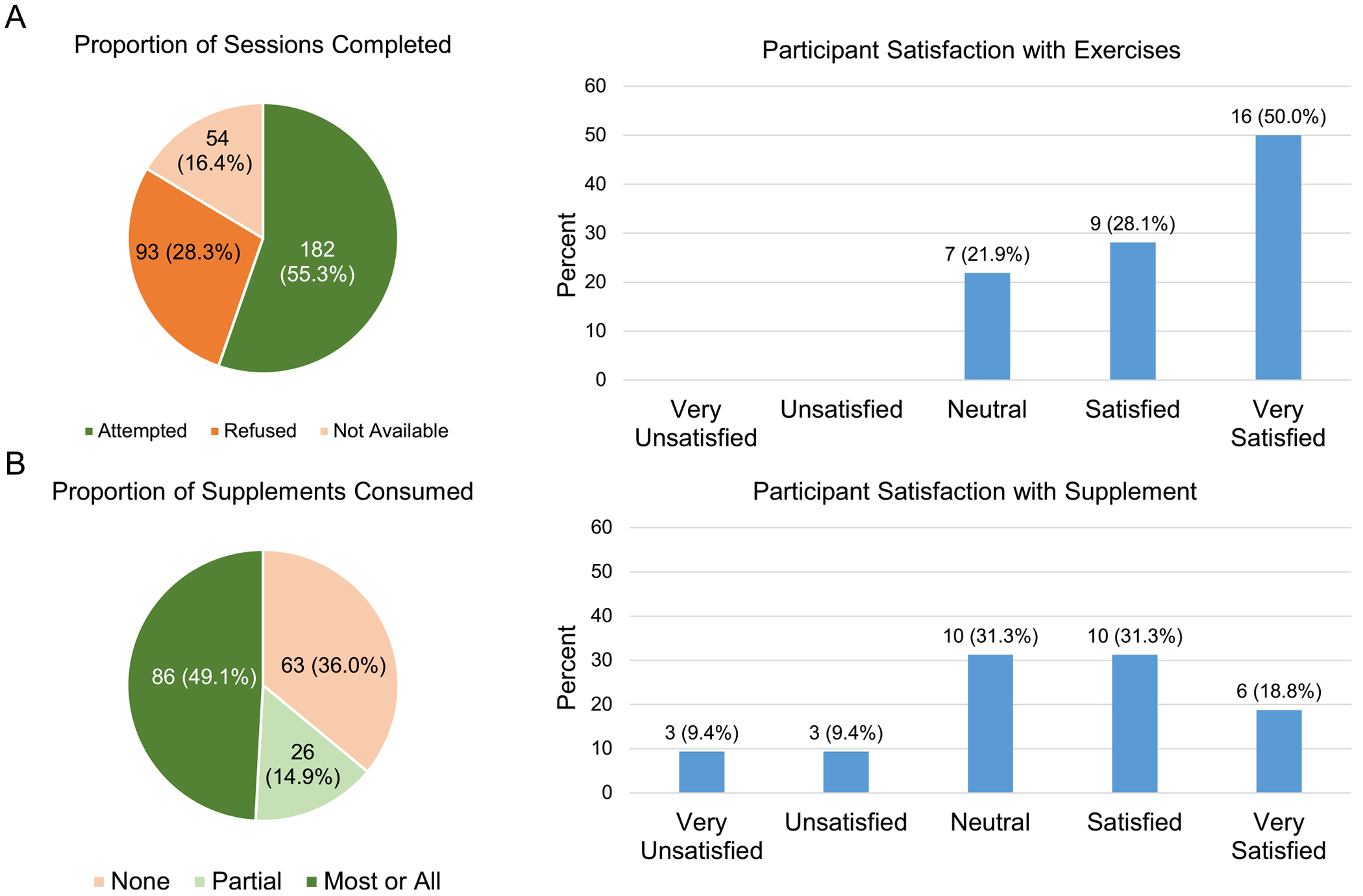
Overall participation and satisfaction among study participants engagement and supplement consumption were reported for all participants regardless of study completion station (*n* = 50). A total of 182 sessions were offered over the duration of the study. Session defined by completion of an offered intervention session. Study participation in sessions was defined as either attempted (participant was offered session and engaged in at least one component), refused (participant declined to participate), or not available (e.g., conflicting medical appointments). The proportion of the supplement consumed was recorded as a percentage and then categorized (0%—None, 25% or 50%—Partial, 75%–100%—Most or All). Satisfaction scores are missing for 16 participants, who did not complete an exit survey either due to hospitalization (8), loss to follow up (1) or study withdrawal (11).

A total of 34 participants completed exit surveys and assessments upon SNF discharge. Overall, 29 (90.6%) of surveyed participants reported being satisfied or very satisfied with the overall program (Figure 2). Among 35 respondents, the Net Promoter Score was 42.9, with 54.3% classified as promoters (scores: 9–10), 34.3% as passives (scores: 7–8), and 11.4% as detractors (scores: 0–6). Of responding participants, 78.1% reported satisfaction with exercises and 50% with supplements. Most participants 21 (58.3%) felt the program was “not too difficult or easy”, with 11 (30.6%) reporting the program was slightly difficult, and only 1 (2.8%) reporting the program was too difficult.

Overall, 22% of study participants withdrew from the study. Of note, the initial rates of withdrawal due to low participation were 36%. In the latter half of the pilot, following the introduction of patient priority care forms, study withdrawal reduced to 8%, with similar rates of session refusal (28.6% vs. 27.9%). Across levels of frailty, there was no difference in participation in intervention sessions (participant refused for prefrail vs. mild vs. moderate or worse: 32.7% vs. 25.6% vs. 29.8%), and cognition (cognitive impairment vs. not: 29.2% vs. 27.6%).

Gait speed could not be obtained for 36 participants (72%) due to medical limitations, including weight-bearing restrictions or safety concerns, and were therefore not analyzed for change. Some functional measures, including PT and OT composite scores, were available from EHR data (*n* = 44). Among participants who completed study discharge assessments, functional outcomes were generally improved from SNF admission to discharge. Mean improvements (Table 3) included: grip strength +1.4 (SD: 3.2), modified Barthel Index +15.9 (SD: 16.8), AMPAC Basic Mobility +3.6 (SD: 4.2), AMPAC Daily Activities +3.1 (SD: 3.3), PT composite score 15.5 (SD: 12.8), and OT composite score 12.2 (SD: 9.3).

## 4 | Discussion

In this pilot study, we demonstrated the feasibility of enrolling patients and delivering a multicomponent frailty intervention in two SNFs. We offered the intervention at least 5 days a week, with overall only 11 (22%) participants withdrawing from the study. More than 90% of surveyed participants were satisfied with the program, and no serious adverse events were reported. These data support the feasibility of recruiting SNF patients across the frailty spectrum, and the tolerability of multicomponent frailty interventions in the post-acute setting.

Our recruitment strategy was designed to identify patients early in the SNF setting without relying on physician documentation or assessment of frailty status. Thus, screening and enrollment were based on the Clinical Frailty Scale. Ultimately, 11 (22%) participants were categorized as prefrail based on the frailty index score, while 38% had moderate frailty or worse, with similar participation across frailty levels. In prior work, even those with prefrailty have benefited from frailty interventions and may be an important target population for future work [41]. Importantly, this demonstrates the feasibility of enrolling even very frail individuals in this intervention. Our recruitment rate of 33% is consistent with prior work in recruiting frail older adults and patients in nursing homes [42], both of which can be particularly challenging for intervention and recruitment [38, 43]. Furthermore, our retention of nearly 80% is quite high, particularly among frail older populations [44]. Future work to explore strategies to include individuals lacking decisional capacity, including those with delirium, as their participation will be critical for generalizability to the broader post-acute SNF population.

The most common reason for exclusion was health care proxy activation, which precluded direct consent for research participation. As this was a novel implementation of a multicomponent (exercise and protein supplement) frailty intervention among frail skilled nursing facility patients, there were concerns regarding participant safety, intervention fidelity, and the potential for harm. Thus, we restricted enrollment to individuals capable of directly providing informed consent for research. Although this somewhat limits the generalizability of our findings, we are crucially able to establish the safety and feasibility of the intervention in frail populations. Future studies should extend this work by adapting and evaluating the intervention among populations with more advanced cognitive impairment or dementia.

Our pilot was limited to two SNFs in the Greater Boston Area. Importantly, all components were delivered without supervision from nurses or rehabilitation professionals. Although the protocol initially planned for PCAs to deliver the intervention, competing clinical responsibilities made it challenging to consistently offer sessions. Many participants requested that sessions be rescheduled or had conflicting medical appointments, requiring several attempts in a single day. To ensure sessions were offered at least 5 days per week, > 90% of sessions were ultimately offered and supervised by research staff. As our intervention demonstrated good safety, with no study-related adverse events, individual supervision during sessions may not be necessary in future work. One limitation is that we did not directly measure participants’ self-efficacy and confidence participating in the intervention. The high proportion of participant satisfaction and an NPS of 42.9% indicates a strong positive endorsement, with several neutral/borderline responses suggesting small improvements could meaningfully improve the program. Altogether, this supports potential scalability and broader dissemination without the need to hire additional rehabilitation or medical professionals for real-world implementation.

Participation was at times limited by fatigue, which contributed to challenges with study retention, particularly during the first half of the trial. Interestingly, participation and feasibility improved substantially after the incorporation of the Patient Priorities Care module into the program. These self-directed modules encouraged participants to reflect on what matters most to them, and to identify personal barriers and facilitators to achieving their goals. Completion of these modules was entirely optional and was completed by only 10 of the 25 participants to whom it was offered. This also corresponded with study withdrawal decreasing from 36% to 8%. Our team observed that some participants were unable to tolerate additional exercise early on in their SNF stay, and thus withdrew after not participating in several sessions. The addition of the priorities module may have allowed for reframing of the intervention within broader care goals. Future work should consider that embedding behavioral and goal-setting strategies may play a critical role in facilitating engagement with rehabilitation interventions in the SNF setting.

As this is a small prospective intervention study, we are also unable to disentangle the effects of the study from standard SNF care, due to the lack of a control group. To some degree, improvement is expected in this setting. Nevertheless, our study demonstrated the feasibility of measuring functional outcomes in this population and highlighted which measures are most responsive to improvement. Some functional outcomes could not be practicably assessed due to medical contraindications, such as weight-bearing status or acute deconditioning after hospitalization. In this regard, the most feasible and consistently captured outcomes were those routinely documented in the SNF setting, including the PT and OT composite score and the modified Barthel index. Because these measures are routinely documented in the EHR, they are pragmatically captured and reduce the risk of missing data bias. Together, these findings suggest that several functional measures are both feasible and sensitive to improvements during the post-acute SNF care. However, further work to explore how these predict long-term functional gains or independence may help guide work to monitor the impact of post-acute SNF care interventions.

In conclusion, a multicomponent frailty intervention was feasible and acceptable in the SNF setting. We successfully recruited participants across a range of cognitive and frailty levels. Further studies will be needed to evaluate broader dissemination and to determine the efficacy of the intervention in improving patient outcomes.

## Supplementary Material

supplemental

Additional supporting information can be found online in the Supporting Information section. Table S1: Exercises selected and frequency of completion. Figure S1: Weekly Participant Exercise Selection Guide. Figure S2: Brief questionnaire to determine capacity for informed consent.

## Figures and Tables

**TABLE 1 |:** Barriers and adaptations to implementation during the pilot study.

Barrier encountered	Protocol adaptations	Observed result
Many potential participants excluded due to admission after ED stay	Removed the requirement that patients be discharged from inpatient hospitalization.	Eight participants enrolled who were admitted after a stay < 3 days in the ED or hospital
Low supplement acceptance due to dietary restrictions (e.g., vegan, kosher) or personal preferences regarding flavors	Broader protein supplement options (including vegan options, protein bars, protein shakes)	Improved satisfaction and consumption of supplements. Participants frequently would explore many options before selecting a preferred one
Patients not available, or sessions not offered consistently by clinical PCAs	Research staff supervised sessions with flexible timing	Consistently able to offer sessions at least 5 days a week
Low participation early in admission	Introduced the self-guided Patient Priorities Care module/workbooks with participants	Improved participant retention. Study withdrawal decreased (36%–8%).

*Note:* Descriptions of adaptations to the protocol to facilitate implementation of the pilot study. Observations and adaptations were not implemented at prespecified endpoints but were introduced on an as-needed basis throughout the study.

Abbreviations: ED, Emergency department; PCA, Patient care associate.

**TABLE 2 |:** Participant baseline characteristics (*N* = 50).

Characteristic	*N* (%)
Age (mean, [SD])	81.2 [7.7]
Female	34 (68%)
White race	43 (86%)
Mean FI (SD)	0.32 (0.08)
Nonfrail (FI < 0.25)	11 (22%)
Mild frailty (FI: 0.25–0.34)	20 (40%)
Moderate frailty (FI: 0.35–0.44)	17 (34%)
Severe frailty (FI ≥ 0.45)	2 (4%)
ADL dependence (0–7, higher is worse)	2.0 (1.6)
IADL dependence (0–7, higher is worse)	3.4 (1.5)
Hospital length of stay days, median [IQR]	6 [4–9]
ICU stay	6 (12%)
Code status	
Full	33 (67%)
Do not intubate	3 (6%)
Do not resuscitate or intubate	13 (27%)
Delirium	3 (6%)
Any fall in the past year	16 (33.3%)
Vision problems	23 (46.0%)
Hearing problems	15 (30.0%)
Polypharmacy (≥ 5 scheduled meds)	47 (94.0%)
Anemia	16 (33.3%)
Cancer history	21 (33.3%)
Diabetes	10 (20.0%)
Hypertension	34 (68.0%)
Pulmonary disease	11 (22.0%)
Heart Failure	12 (24.0%)
Coronary artery disease	14 (28.0%)
Stroke/transient ischemic attack	9 (24.0%)
Depression	13 (27.7%)
Anxiety	16 (33.3%)
Dementia	4 (8.0%)
Prescribed benzodiazepines	7 (14.0%)
Prescribed antipsychotics	3 (6.0%)
Prescribed opioids	23 (46.0%)

*Note:* Frailty status, ADLs, and IADLs reflect prehospitalization status. Code status and delirium reflect status on SNF admission, with delirium defined by 3D-CAM assessment positivity on admission assessment. Medication prescriptions determined from the SNF electronic health record.

Abbreviations: ADL, Activities of Daily Living; FI, Frailty Index; IADL, Instrumental Activities of Daily Living; IQR, Interquartile Range; SD, Standard Deviation.

**TABLE 3 |:** Observed changes in functional outcome scores over SNF stay.

	Baseline function *n* = 50 mean (SD)	Completer baseline function *n* = 34 mean (SD)	Discharge function *n* = 34 mean (SD)	Mean difference
Grip strength (kg)	15.4 (6.9)	15.8 (8.0)	17.4 (8.4)	1.4 (3.2)
Modified barthel index	53.3 (16.6)	55.1 (15.2)	71.4 (18.0)	15.9 (16.8)
AM-PAC basic mobility	12.2 (2.8)	12.9 (2.6)	16.7 (3.6)	3.7 (4.2)
AM-PAC ADLs	15.9 (3.4)	16.7 (3.1)	19.7 (3.0)	3.1 (3.3)
PT composite score	29.4 (9.7)	28.8 (9.9)	44.6 (12.1)	15.5 (12.8)
OT composite score	27.0 (7.1)	27.6 (7.3)	39.1 (11.0)	12.2 (9.3)
PROMIS physical health	33.5 (5.9)	33.2 (5.2)	37.4 (7.0)	3.7 (7.3)
PROMIS mental health	44.9 (8.0)	44.8 (8.1)	46.8 (8.3)	1.7 (6.8)

*Note: n* = 44 for grip strength at baseline and 29 on discharge. PT and OT composite discharge score *n* = 44 from electronic health records. Otherwise, discharge function scores are missing for 16 participants due to acute re-hospitalization, loss to follow-up, or study withdrawal. Mean difference is only calculated among those who completed the study.

Abbreviations: ADLs, Activities of daily living; AM-PAC, Activity measure for post-acute care; OT, Occupational therapy; PROMIS, Patient reported outcome measurement information system; PT, Physical therapy; SNF, Skilled nursing facility.

## Summary

- Key Points
  - A multicomponent frailty intervention is safe, feasible, and acceptable to older adults receiving post-acute care
  - Older adults with frailty safely tolerate exercise and protein supplement in addition to usual care in skilled nursing facilities.
- Why does this paper matter?
  - This represents a promising strategy to improve post-acute SNF outcomes among this population.
