# Supplementary material for: Feasibility of a Multicomponent Frailty Intervention During Post-Acute Rehabilitation in Skilled Nursing Facilities
Source: J Am Geriatr Soc. Author manuscript; Available in PMC 2026 Jun 7. (PMC13242210; doi:10.1111/jgs.70497)
Supplement: supplemental [file NIHMS2175559-supplement-supplemental.pdf]

Supplemental Materials

Supplemental Table 1. Exercises selected and frequency of completion
Supplemental Figure 1. Weekly Participant Exercise Selection Guide
Supplemental Figure 2. Brief Questionnaire to Determine Capacity for Informed Consent

Supplemental Table 1. Frequency of Exercise Recommendation and Completion

**Exercise**	**Recommendation Count (%)**	**Completed Sessions Count (%)**
Walking	18 (20.5%)	28 (8.5%)
Standing Marching	14 (15.9%)	22 (6.7%)
Sitting to Standing	17 (19.3%)	16 (4.9%)
Chair Push-Up	25 (28.4%)	30 (9.1%)
Sitting Quad Set	62 (70.5%)	121 (36.8%)
Triceps Pull Down	34 (38.6%)	79 (24.0%)
Shoulder Retraction / Reverse Fly	40 (45.5%)	97 (29.5%)

Percentages reflect the proportion of times each exercise was selected. Exercise recommendations were assessed by calculating the number of times a set was selected, given the total number of exercise prescriptions done in the entire study(N = 88). Exercise completion was assessed across all intervention sessions for the entire study (N = 329). Participants had to complete all sets with all repetitions to count as completion. Percentages, therefore, should not be interpreted as conditional completion among those prescribed a given exercise.

Supplemental Figure 1. Weekly Participant Exercise Selection Guide

Name:___________ Please select up to 3 exercises Date:__________

A. Walking

Can use assistive device *and* gait belt

Distance: _____________

(to Window, End of Hall)

(est. _____________ ft)

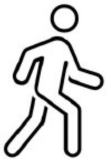

B. Standing Marching

March in place by lifting left leg up, then right. Alternate legs.

(Can use support)

_____ Reps each set

_____ Sets in total

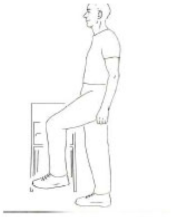

C. Sitting to Standing

With straight back, tighten stomach, place dominant leg back under chair, lean slightly forward and stand up.

_____ Reps each set

_____ Sets in total

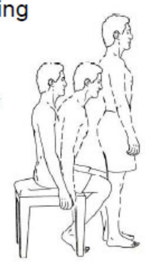

D. Shoulder Retraction

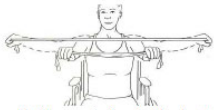

Extend both arms in front of body at shoulder height, palms down, holding band. Move arms out to sides, squeeze shoulder blades together.

_____ Reps each set

_____ Sets in total

E. Triceps Pull Down

In shoulder width stance, anchor band over head with extended arm. Pull other arm down, straightening elbow

_____ Reps each set

_____ Sets in total

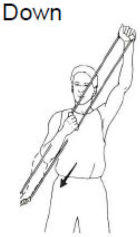

Theraband resistance/color:___________

F. Chair Push Up

Lift buttocks off seat of chair by pushing down with arms

_____ Reps each set

_____ Sets in total

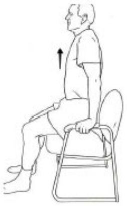

G. Sitting Quad Set

Tighten muscle in top of thigh and straighten out knee. Hold 3 seconds while counting out loud. Keep thigh on chair. Repeat with other leg.

_____ Reps each set

_____ Sets in total

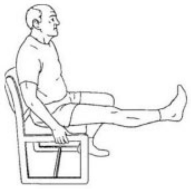

Supplemental Figure 2. Brief Questionnaire to Determine Capacity for Informed Consent

We would like to verify your understanding of what the study is about and of your rights as a study participant. This is a part of our standard procedure.

<u>Ability to appreciate the situation and its likely consequences</u>

**1. Is the research study separate from the care you are getting here at [STUDY SITE]?**<br>Patient understands the difference from research and clinical care.	Correct	Incorrect
**2. If you do not participate in the study, will it affect your medical care?**<br>Patient understands that declining participation will not affect medical care	Correct	Incorrect
**3. Will the information you give us for this study be kept secret and confidential within the study personnel and authorized staff?**<br>Patient understands that all their information will be kept confidential.	Correct	Incorrect

<u>Ability to evidence a choice:</u>

**4. Can you quit the study any time after you have agreed to participate?**<br>Patient is aware that he/she can discontinue study participation at any time.	Correct	Incorrect

<u>Overall capacity to provide informed consent</u>:

Total # of “Incorrect”: __/4 If “Incorrect” for 2 or more questions, **or only Question 4 is incorrect**, they cannot consent for the study.
